# Mechanistic assessment of the analgesic, anti-inflammatory and antipyretic actions of *Dalbergia saxatilis* in animal models

**DOI:** 10.1080/13880209.2017.1283706

**Published:** 2017-02-01

**Authors:** Omoniyi K. Yemitan, Olufunmilayo O. Adeyemi

**Affiliations:** a Department of Pharmacology, Lagos State University College of Medicine, Ikeja, Lagos, Nigeria;; b Department of Pharmacology, Therapeutics and Toxicology, College of Medicine of the University of Lagos, Idi-Araba, Lagos, Lagos, Nigeria

**Keywords:** Writhing test, formalin test, dextran, carrageenan, amphetamine, yohimbine, turpentine, lipopolysaccharide, phenolic tannins

## Abstract

**Context:** Aqueous root extract of *Dalbergia saxatilis*, Hook, f., (Leguminosae) (DS) is reported useful for toothache, pains, and fever, but not scientifically proven.

**Objective:** This study determined its effectiveness in pain, inflammation, and fever, applying scientific models.

**Materials and methods:** Swiss mice or Sprague–Dawley rats (*n* = 5) were pretreated with distilled water, DS (100 or 200 mg/kg), or standard drug for 30 min. The analgesic activity was measured by acetic acid writhing, tail flick, tail immersion, tail clip, hot plate, and formalin pain tests; anti-inflammatory effects were determined via carrageenan and dextran rat paw oedema tests; antipyretic activity was measured by *Escherichia coli* lipopolysaccharide (ECL) and turpentine in rabbits, and d-amphetamine sulphate (d-AS) pyrexia test in rats.

**Results:** Writhing frequency inhibition was produced by 200 mg/kg DS (33.10%), aspirin (38.19%) and morphine (93.68%). Unlike morphine, DS did not produce significant prolongation of the reaction times in the hot-plate, tail immersion, tail flick, and tail clip tests. In the first and second phases of formalin test, respectively, % inhibition was: 200 mg/kg DS (25.70% and 0%), aspirin (4.76% and 67.33%), morphine (81.42% and 66.11%); for carrageenan and dextran tests, significant difference was recorded between 200 mg/kg DS and control up to 6 h. Significant reduction in ECL, turpentine and d-AS pyrexia was recorded at 100 and 200 mg/kg DS.

**Conclusion:** DS produces mild non-steroidal analgesic and anti-inflammatory, as well as significant antipyretic actions involving cyclooxygenase, α_2_ adrenoceptor and interleukin-1 β1 due to any of glycosides, saponins or phenolic tannins.

## Introduction

Many people, both in rural and urban areas of the world are now turning to herbal products for various reasons, including low cost and easier accessibility (Vann 1998; Vandebroek et al. 2004). Personal expenditures on herbal medicines has been estimated to amount to 31,000,000£ in the United Kingdom (Thomas et al. 2001), and approximately $5,000,000,000 in the United States. Moreover, reports indicate that, not less than one-third of the world population uses herbal alternatives (Barnes et al. 2004). Herbal therapies are more likely to be used by those with better education, poorer health status, and a holistic orientation to health, those wanting relief from symptoms or seeking improvement in their general condition, and those who had a transformational experience that changed their world view (Kimby et al. 2003). According to a World Health Organization (WHO) report (Aschwanden 2001), in Africa, up to 80% of the population depends on herbs; in India, Canada, and France, 65%, 50% and 75% of the population, respectively, use herbal alternatives alone or to supplement orthodox pharmaceuticals; in Japan, 85% of doctors prescribe not only modern medicine, but also the traditional herbal medicine (Aschwanden 2001). Currently, WHO advocates the inclusion of herbal medicine in primary health care because of their great potential (Akuodor et al. 2015).

Roughly one-quarter to one-half of current pharmaceuticals were originally obtained from plants (Ernst & Pittler 1998), of which analgesic, anti-inflammatory, and antipyretic drugs are sizeable. Examples include poppy herb (morphine), white willow tree bark (salicin), and cinchona bark (quinine). Pain, inflammation and fever will forever remain physiological responses to, or symptoms of a variety of ailments, sicknesses, and diseases afflicting humanity. Therefore, the search for sources of these important drug agents outside the current synthetic ones cannot be dismissed. Moreover, the ideal analgesic, anti-inflammatory, and antipyretic agents in terms of efficacy, potency, safety and cost, are yet to be discovered, especially from plant sources.

*Dalbergia saxatilis* Hook, f. (Leguminosae; sub family: Papilionaceae) is a woody shrub widely distributed in the forest and savannah regions of Africa especially West Africa, where the plant parts are employed for various medicinal uses (Gill 1992). Herbalists in some areas of Nigeria have for a long time, traditionally employed the root decoction of the plant in the treatment of toothache and abdominal pain, among other uses (Oliver 1960).

Similarly, the various classes of synthetic analgesic, anti-inflammatory and antipyretic drugs in use today are effective but not without adverse effects (Gris et al. 2010).

The analgesic, anti-inflammatory and antipyretic activities of the methanol leaf extract of *D. saxatilis* has now been reported (Ismail et al. 2015); but based on the traditional uses of the root extract of *Dalbergia saxatilis*, its aqueous extract was used to assess the analgesic, anti-inflammatory, and antipyretic effects as well as possible mechanisms of action, applying various animal models.

## Materials and methods

### Plant material

Fresh roots of *Dalbergia saxatilis* were collected in May, 2007, from secondary forests in Ikire, Osun State, Nigeria. The material was authenticated by T.K. Odewo, who was then a senior superintendent of the Forestry Research Institute of Nigeria (FRIN), where a voucher specimen (FHI 106484) was deposited for reference; confirmation was done by Professor J.D. Olowokudejo of the Botany and Microbiology Department, University of Lagos, Nigeria.

### Extract preparation

The root of the freshly harvested *D. saxatilis* were dried and ground into powdered form, then boiled in distilled water (100 g/L) for 30 min. It was then left for 24 h at room temperature for further extraction and filtered. The filtrate was evaporated to dryness in an oven set at 40 °C, with extract yield: 12.6 ± 1.3% (range: 11–14%) of weight of the dried root. The dried extract was stored in the refrigeration at 2 °C until ready for use. Administration was done by dissolving the extract in distilled water with a stock solution of 500 mg/mL concentration. Final administration to each animal was calculated such that each animal was administered with extract solution of not more than 0.1 mL/10 g of a mouse, 0.4 mL/100 g of a rat, or 1 mL/kg of rabbit. Standardization of the extract was ensured by following the same protocol expressed above during the extraction and administration.

Preliminary phytochemical screening of DS revealed the presence of glycosides, saponins, and phenolic tannins. Moreover, a bioactive triterpenoid has also been reported to be isolated from the root (Uchendu & Leek 1999; Uchendu et al. 2000), isolated as betulinic acid (Koma & Sani 2014).

### Animals

The animals used for the experiments were adult Swiss albino mice of both sexes (20–25 g), adult male Sprague–Dawley rats (180–210 g), or male rabbits (3.2–3.6 kg) which were obtained from, and kept at the Laboratory Animal Centre of the College of Medicine of the University of Lagos, Idi-Araba, Lagos, Nigeria. The animals were maintained under standard environmental conditions, being fed with standard Pfizer-branded rodent feeds obtained from Livestock Feeds, Nigeria Ltd. and given water *ad libitum*. All animals were kept at room temperature in cross-ventilated rooms, without illumination at night to achieve 12 h light/dark periods. The animals were acclimatized in the laboratory condition for 14 days prior to the experiment, during which they were given free access to food and water *ad libitum*. The care and use of animals were conducted in accordance with the National Institute of Health Guide for the Care and Use of Laboratory Animals (NIH 1996). Moreover, Ethical approval for animal use was obtained from the Experimental Ethics Committee on Animal Use of the College of Medicine of the University of Lagos, Idi-Araba, Lagos, Nigeria.

### Doses selection for *D. saxatilis*

Based on preliminary tests in our laboratory, the minimum and maximum effective doses of the aqueous root extract of *D. saxatilis* were 100 and 200 mg/kg for analgesia, anti-inflammatory, and antipyretic (except for amphetamine study in which antipyretic response was only achieved at 200 mg/kg of *D. saxatilis*) effects. Above these doses, no better efficacy of response was recorded. Furthermore, an earlier study by Yemitan and Adeyemi (2003) revealed that DS did not produce any mortality up to 5000 mg/kg, orally in mice. These were the bases for the doses selection in this study.

### Analgesic study

#### Acetic acid*-*induced writhing test

This was based on the method described by Koster et al. (1959). Mice of either sex were divided into five groups of five animals each. Distilled water (10 mL/kg, p.o., control), Aqueous root extract of *Dalbergia saxatilis*, DS (100 & 200 mg/kg, p.o.), or aspirin (100 mg/kg, p.o.) was administered to each group 60 min before i.p. injection of 10 mL/kg, 0.6% v/v glacial acetic acid solution. The number of writhes (a syndrome characterized by a wave of contraction of abdominal musculature followed by extension of hind-limb) was counted for 30 minutes. A reduction in the number of writhes indicated analgesic property (Surender & Majumdar 1995). Percentage inhibition data were calculated and reported according to the following formula:

$$\%\mathrm{Inhibition}\;\;=\;\frac{\text{Mean number of writhes}\left(\mathrm{Control}\right)-\text{Mean number of writhes }\left(\mathrm{Test}\right)}{\text{Mean number of writhes }\left(\mathrm{Control}\right)}\;\;\times \;100$$

#### Tail flick test

The rat cold*-*water tail flick assay was based on modification (Clark et al. 1988) of the method originally described by Pizziketti et al. (1985). Each rat was closely restrained in a wire mesh cage and the lower half of its tail dipped in a beaker of cold water (0–1 °C). The time in seconds for tail withdrawal from the water was taken as the reaction time. Measurement of threshold was done before, and at 30 min. interval after administration of distilled water (10 mL/kg, p.o.), DS (100 and 200 mg/kg, p.o.), or morphine (10 mg/kg, i.p.) to different groups (*n* = 5).

#### Tail immersion test

The lower 5 cm portion of the rat tail was dipped in a hot water bath maintained at 55.0 ± 0.5 °C (Janssen et al. 1963; Adeyemi et al. 2005). The time in seconds to clearly withdraw the tail out of the water was taken as the reaction time. Reaction times were taken immediately after administration of the extract and at 30, 60, 90, 120 and 150 min after administration of distilled water (10 mL/kg, p.o.), DS (100 and 200 mg/kg, p.o), or morphine (10 mg/kg, i.p.) to different groups (*n* = 5).

#### Tail clip test

Mice were screened by applying a metal artery clip to the base of the tail with its jaw sheathed with thin rubber tubing. The animals that did not attempt to dislodge the clip within 10 s were discarded. The responsive mice were allotted to groups of five animals each. The tail clip was applied 30, 60, 90, 120 and 150 min after oral administration of distilled water (10 mL/kg, p.o.), DS (100 and 200 mg/kg, p.o.), or morphine (10 mg/kg, s.c.), (*n* = 5). Analgesic property was considered to be present if no attempt was made to dislodge the clip within 10 s (Surender & Majumdar 1995).

#### Hot plate test

The heated surface of a hot plate was maintained at 55.0 ± 0.5 °C. Each young adult albino mouse was gently placed on the plate and the time required for the mouse for paw-licking or jumping was taken as the response (Wolff & MacDonald 1994) and to avoid tissue damage the cutoff time or latency response in the control was taken as 15 s. The test was performed before administration of distilled water (10 mL/kg, p.o.), DS (100 and 200 mg/kg, p.o.), or morphine (10 mg/kg, i.p.) (*n* = 5) and was repeated at 30 min interval until 150 min.

#### Formalin test

The method used was similar to that described previously (Shibata et al. 1980; Viana et al. 1998). Formalin (1%, 20 μL) was injected subcutaneously into the right hind paw of mice. The time (in seconds) spent for licking and biting responses of the injected paw was taken as an indicator of pain response. Responses were measured for 5 min after formalin injection (first phase) and 15–30 min after formalin injection (second phase). The extract (100 and 200 mg/kg, p.o), aspirin (100 mg/kg, p.o.), morphine (10 mg/kg, s.c.) or distilled water (10 mL/kg, p.o.) (*n* = 5) was administered 30 min before formalin injection. A reduction in the number of paw licking is indicative of analgesic property (Sugimoto et al. 1986). 

### Anti-inflammatory study

#### Carrageenan-induced rat paw oedema test

Young adult albino rats of both sexes were fasted for 12 h and randomly allotted to five groups of six animals (3/sex) each. Different groups of rats were pretreated with extract (100 and 200 mg/kg, p.o.) or indomethacin (10 mg/kg, s.c.). The control group received normal saline (10 mL/kg, p.o.). Thirty minutes, later, oedema was induced by injecting carrageenan (0.1 mL, 1% w/v in normal saline) into the sub-plantar tissue of the right hind paw and 0.1 mL normal saline into the left hind paw of each animal (Winter et al. 1962). The linear paw circumference was measured using the white cotton thread method (Bamgbose & Noamesi 1981) immediately at 0 h, 0.5 h and hourly for 5 h after carrageenan injection. The differences in the left and right paw volumes indicated the degree of inflammation.

#### Dextran-induced rat paw edema test

Young adult albino rats of both sexes were fasted for 12 h and randomly allotted to five groups of six animals each Different groups of rats were pretreated with DS (100 and 200 mg/kg, p.o.) or indomethacin (10 mg/kg, s.c.). The control group received normal saline (10 mL/kg, p.o.). Thirty minutes, later, inflammatory edema was induced by injecting dextran (0.1 mL, 1% w/v in normal saline) into the sub-plantar tissue of the right hind paw and 0.1 mL normal saline, into the left hind paw of each animal (Winter et al. 1962). The linear paw circumference was measured using the white cotton thread method (Bamgbose & Noamesi 1981) immediately at 0 h, 1 h, 3 h and 6 h after dextran injection. The differences in the left and right paw volumes indicated the degree of inflammation.

#### Antipyretic study

Four different agents were used to induce pyrexia; namely, lipopolyssacharide of *Escherichia coli* (ECL), 2,4–dinitrophenol (2,4–DNP), d-amphetamine sulfate (d-AS) and turpentine solvent (TS). Except otherwise stated, the control group of animals were administered with 10 mL/kg normal saline.

#### *Escherichia coli* lipopolyssacharide-induced pyrexia test

Each rabbit, fasted for 12 h, was restrained in a holder, and allowed to stabilize for some minutes after which a basal rectal temperature was taken using a narrow bulb digital rectal thermometer. Pyrexia was induced by intravenous injection of lipopolysaccharide obtained from *E. coli*, through the marginal ear vein (Vogel & Vogel 2005). Different groups of rabbit or rat (*n* = 5) were pretreated with the extract (100 and 200 mg/kg, p.o.), aspirin (100 mg/kg, p.o.) or normal saline (10 mL/kg, p.o.). The rectal temperature of each rabbit was then measured, immediately (0 min), 0.5 h and at 1 h interval, until the temperature was constant or there was a drop in the temperature of the control animal. Unless stated otherwise, the same dosing schedule and procedure, as above, was used for measurements of antipyretic effects in each model.

#### Turpentine-induced pyrexia test

Pyrexia was induced in rabbits by intravenous injection of 0.2 mL/kg of turpentine solvent through the marginal ear vein. Maximum hyperthermia was observed 30 min after turpentine was injected in control animals (*n* = 5). It was a modification of the method of Petrova et al. (1978).

#### d-Amphetamine sulphate-induced pyrexia test

To carefully-selected docile rats, yohimbine HCl (Sigma, 10 mg/kg) was administered subcutaneously to one of the groups for 60 min. before d-AS. Pyrexia was induced by i.p. injection of 10 mg/kg d-AS to rats (Berken et al. 1991; Blackhouse et al. 1994; Mantegazza et al. 1994). Maximum hyperthermia was observed 1 h after d-AS was injected in control animals (*n* = 5).

### Statistical analysis

Results are presented as mean ± S.E.M or percentages. Statistical significance between the groups was analyzed by means of analysis of variance (ANOVA) followed by Fisher’s *post-hoc* PLSD multiple comparison tests. *p* values less than 0.05 or 0.01 were considered significant.

## Results

### Analgesic effect of *D. saxatilis*

#### Acetic acid-induced writhing test

A significant (*p* < 0.05) reduction in the number of writhes was produced by oral doses of extract (100 and 200 mg/kg). At 200 mg/kg, % inhibition of writhes was comparable to that of aspirin (100 mg/kg, p.o.), but significantly lower than that of 10 mg/kg, morphine (Table 1).

**Table 1.** Effect of *D. saxatilis* on acetic acid-induced writhing in mice.

Treatment	Dose (mg/kg)	No of writhing (per 15 min)	% Inhibition
Control	–	72.8 ± 5.7	–
*D. saxatilis*	100	56.5 ± 4.9^a^^,^^b^^,^^c^	22.39
	200	48.7 ± 4.1^a^^,^^b^	33.10
Aspirin	100	45.0 ± 4.3^a^^,^^b^	38.19
Morphine	10	4.6 ± 0.4^a^^,^^c^	93.68

Table showing the effects on writhing of *Dalbergia saxatilis,* aspirin, morphine or distilled water administered 30 min before intraperitoneal acetic acid in mice. Each value represents mean ± S.E.M or percentage inhibition of pain as compared to control animals, *n* = 5. Significant at *p* < 0.05 (ANOVA, Fisher’s *PLSD* test);

a Significant inhibition compared to control given distilled water.

b Significantly different inhibition compared to morphine-treated.

c Significantly different inhibition compared to aspirin-treated.

#### Tail flick test

Unlike morphine, the extract did not produce any significant prolongation or inhibition of the reaction time to tail flick (Table 2).

**Table 2.** Effect of *D. saxatilis* on tail flick test in rats.

Treatment	Dose (mg/kg)	Pretreatment time to tail flick (s)	Post-treatment time to tail flick (s)	% Inhibition
Control	–	18.1 ± 1.2	18.7 ± 2.4	–
*D. saxatilis*	100	19.1 ± 1.3	19.9 ± 3.1^b^	1.2
	200	18.9 ± 2.1	20.0 ± 3.2^b^	1.7
Morphine	10	17.7 ± 2.3	53.3 ± 5.1^a^	65.6

Table showing the effects of pretreatment with *Dalbergia saxatilis,* morphine or distilled water at intervals of 30 min on the tail flick test in rats whose tails were dipped in ice (0–1 °C). Each value represents mean ± S.E.M or percentage inhibition of pain as compared to control animals, *n* = 5. Significant at *p* < 0.05 (ANOVA, Fisher’s *PLSD* test).

a Significant inhibition compared to control given distilled water;

b Significantly lower inhibition compared to morphine-treated.

#### Tail immersion test

Unlike morphine, the extract produced neither significant prolongation nor inhibition of the reaction time to tail withdrawal from hot water (Table 3).

**Table 3.** Effect of *D. saxatilis* on tail immersion test in rats.

Treatment	Dose (mg/kg)	Pretreatment timeto tail withdrawal (s)	Post-treatment timeto tail withdrawal (s)	% Inhibition
Control	–	1.9 ± 0.2	1.9 ± 0.3	–
*D. saxatilis*	100	1.9 ± 0.1	2.1 ± 0.5^b^	1.9
	200	2.0 ± 0.2	2.3 ± 0.4^b^	2.6
Morphine	10	2.0 ± 0.1	7.2 ± 0.7^a^	71.4

Table showing the effects of pretreatment with *Dalbergia saxatilis,* morphine or distilled water at intervals of 30 min on tail withdrawal in rats whose tails were dipped in hot water (55.0 ± 0.5 °C). Each value represents mean ± S.E.M or percentage inhibition of pain as compared to control animals, *n* = 5. Significant at *p* < 0.05 (ANOVA, Fisher’s *PLSD* test),

a Significant inhibition compared to control given distilled water;

b Significantly lower inhibition compared to morphine-treated animals.

#### Tail clip test

Unlike morphine, the extract produced neither significant prolongation nor inhibition of the reaction time to attempt to dislodge clip from tail (Table 4).

**Table 4.** Effect of *D. saxatilis* on tail clip test in mice.

Treatment	Dose (mg/kg)	Pretreatment reaction time (s)	Post-treatment reaction time (s)	% Inhibition
Control	–	3.17 ± 0.23	3.23 ± 0.30	–
*D. saxatilis*	100	3.21 ± 0.26	3.26 ± 0.41^b^	1.3
	200	3.14 ± 0.23	3.26 ± 0.36^b^	2.8
Morphine	10	3.19 ± 0.27	10.39 ± 0.58^a^	77.2

Table showing the effects of *Dalbergia saxatilis,* morphine or distilled water after 30 min on the attempt to dislodge a clip at the base of tail of mouse. Each value represents mean ± S.E.M or percentage inhibition of pain compared to control, *n* = 5. Significant at *p* < 0.05, (ANOVA, Fisher’s *PLSD* test),

a Significant inhibition compared to control given distilled water.

b Significantly lower inhibition compared to morphine-treated animals.

#### Hot plate test

Unlike morphine, the extract did not produce any significant prolongation of the reaction time to paw licking or attempt to jump from the hot plate (Table 5).

**Table 5.** Effect of *D. saxatilis* on hot-plate test in mice.

Treatment	Dose (mg/kg)	Pretreatment reaction time (s)	Post-treatment reaction time (s)	% Inhibition
Control	–	2.32 ± 0.37	2.31 ± 0.39	–
*D. saxatilis*	100	2.23 ± 0.43	2.28 ± 0.40^b^	1.7
	200	2.47 ± 0.51	2.63 ± 0.51^b^	1.7
morphine	10	2.42 ± 0.46	11.68 ± 0.59^a^	68.45

Table showing the paw-licking or jumping activity of mice placed on a hot plate (55.0 ± 0.5 °C), 30 min after pretreatment with *Dalbergia saxatilis,* morphine or distilled water. Values represent mean ± S.E.M or percentage inhibition of pain compared to control, *n* = 5. Significant at *p* < 0.05 (ANOVA, Fisher’s *PLSD* test),

a Significant inhibition compared to control given distilled water;

b Significantly lower inhibition compared to morphine-treated animals.

### Formalin test

The extract, at 200 mg/kg, unlike aspirin, produced a significant reduction in paw-licking in the first phase; but unlike morphine or aspirin, did not inhibit paw-licking time in the second phase of formalin-induced pain test (Table 6).

**Table 6.** Effect of *D. saxatilis* on formalin test in mice.

Treatment	Dose (mg/kg)	Total paw-licking time first phase (0-5 min) (s)	% Inhibitionfirst phase	Total paw-licking time second phase (15-30 min) (s)	% Inhibitionsecond phase
Control	–	109.0 ± 4.2	–	103.4 ± 5.4	–
*D. saxatilis*	100	100.2 ± 4.9^b^^,^^c^	4.92^b^^,^^c^	106.6 ± 4.3^b^^,^^c^	0^b^^,^^c^
	200	81.6 ± 4.8^a^^,^^b^	25.70^a^^,^^b^	100.3 ± 4.6^b^^,^^c^	0^b^^,^^c^
Aspirin	100	99.8 ± 4.5^b^	4.76^b^	64.8 ± 4.6^a^	67.33^a^
Morphine	10	22.8 ± 2.5^a^^,^^c^	81.42^a^^,^^c^	64.3 ± 3.9^a^	66.11^a^

Table showing the hindpaw-licking activity of mice injected with formalin after pretreatment with *Dalbergia saxatilis,* aspirin, morphine or distilled water. Each value represents mean ± S.E.M or percentage inhibition of pain compared to control, *n* = 5. Significant at *p* < 0.05 (ANOVA, Fisher’s *PLSD* test),

a Significant inhibition compared to control given distilled water;

b Significantly different inhibition compared to morphine-treated animals;

c Significantly different inhibition compared to aspirin-treated animals.

### Anti-inflammatory effects of *D. saxatilis*

#### Carrageenan-induced rat paw oedema

**Figure 1.**
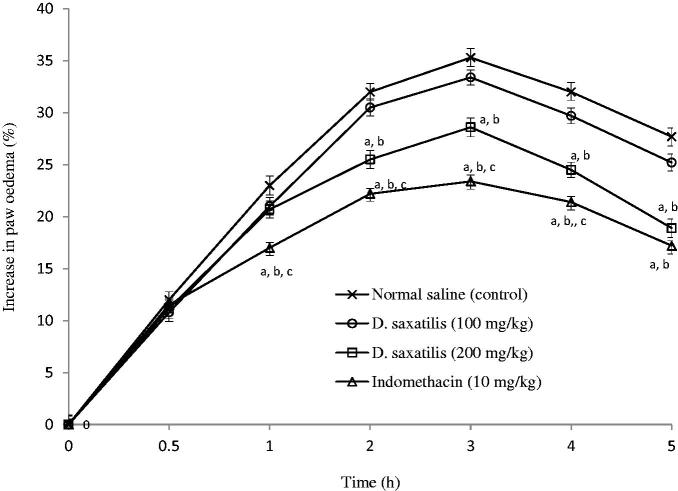
Graphs showing mean increases in paw oedema with time after normal saline (10 ml/kg), *D. saxatilis* (100 and 200 mg/kg) or indomethacin was administered into mice before carrageenan was injected into the right hind paw of rats (*n* = 6 per group). Significant (*p* < 0.05; ANOVA, Fisher’s *PLSD* test) reduction in oedema formation compared with ^a^control given normal saline; ^b^100 mg/kg *D. saxatilis;*^c^200 mg/kg *D. saxatilis*-treated animals at the control at the same time.

Significant (*p* < 0.05; Student’s *t*-test) reduction in paw volume between DS at 200 mg/kg and control was recorded up to the end of second hour. However, indomethacin pretreatment produced significant lower oedema formation in rats compared to the extract (Figure 1).

#### Dextran-induced rat paw edema

**Figure 2.**
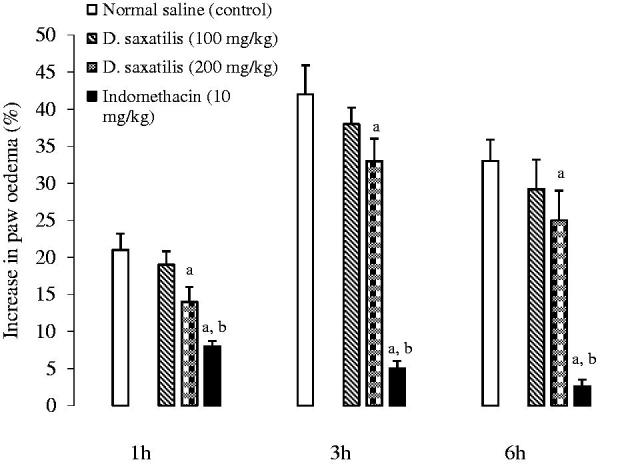
Charts showing mean increases in paw oedema with time after normal saline (10 ml/kg), *D. saxatilis* (100 and 200 mg/kg) or indomethacin (10 mg/kg, s.c.) was administered into mice before dextran was injected into the right hind paw of rats (*n* = 6 per group). ^a^Significant (*p* < 0.05; ANOVA, Fisher’s *PLSD* test) reduction in oedema formation compared with the control; ^b^Significant (*p* < 0.05; ANOVA, Fisher’s *PLSD* test) reduction in oedema formation compared with the extract, at the same time.

At 1 h post-dextran treatment, unlike indomethacin which produced significant reduction in oedema, there were no significant differences between the paw enema in the 100 and 200 mg/kg extract-treated rats and control. However, at 3 h and 6 h post-treatment, there were reductions in paw oedema, reaching statistical significance (*p* < 0.05) at 200 mg/kg of extract compared with the control. Indomethacin pretreatment, however, produced more significant (Student’s *t*-test) reduction in oedema formation in rats compared to the extract (Figure 2).

### Antipyretic effects of *D. saxatilis*

#### *Escherichia coli* lipopolysaccharide-induced pyrexia

**Figure 3.**
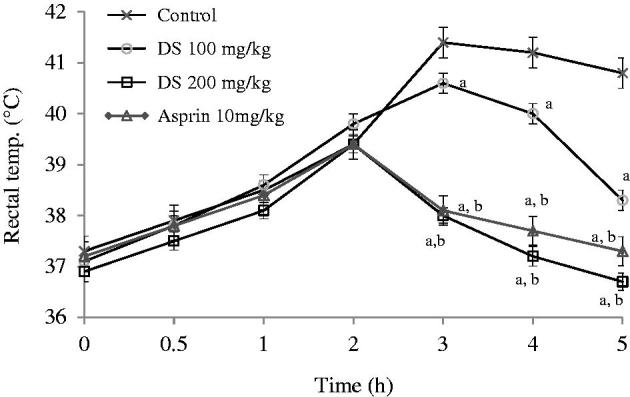
Graphs showing the effects of control (normal saline, 10 ml/kg), aqueous root extract of *Dalbergia saxatilis*, DS (100 mg/kg and 200 mg/kg) and aspirin (100 mg/kg) on pyrexia induced by *E. coli lipopolysaccharide* in rabbits. Significant (*p* < 0.05; ANOVA, Fisher’s *PLSD* test) reduction in rectal temperature compared with ^a^control given normal saline; ^b^100 mg/kg *D. saxatilis;*^c^200 mg/kg *D. saxatilis*-treated animals at the same time; *n* = 5 per group.

Maximum pyrexia was observed 3 h after *E. coli* lipopolysaccharide (ECL) was injected in control animals. Extract (200 mg/kg) produced a significant (*p* < 0.05) reduction in pyrexia induced by ECL. This effect was produced after 3 h and comparable to that produced by aspirin (Figure 3).

#### Turpentine-induced pyrexia

**Figure 4.**
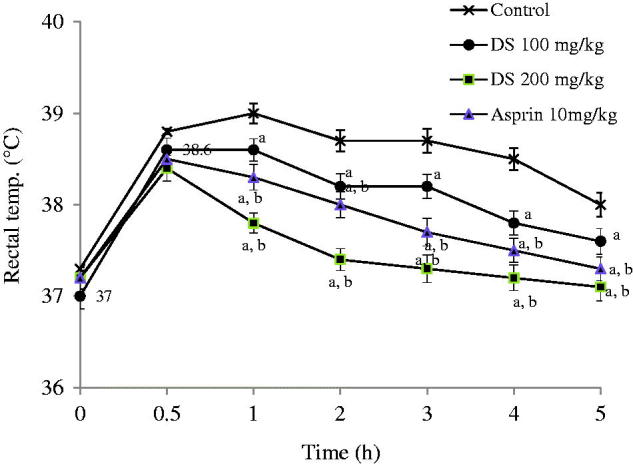
Graphs showing the effects of control (normal saline, 10 ml/kg), aqueous root extract of *Dalbergia saxatilis*, DS (100 mg/kg and 200 mg/kg) and aspirin (100 mg/kg) on pyrexia induced by turpentine solvent in rabbits. Significant (*p* < 0.05; ANOVA, Fisher’s *PLSD* test) reduction in rectal temperature compared with ^a^ control given normal saline; ^b^ 100 mg/kg *D. saxatilis;*^c^ 200 mg/kg *D. saxatilis*-treated animals at the same time. (*n* = 5 per group).

Maximum pyrexia was observed 1 h after turpentine was injected in control animals. The extract (100 and 200 mg/kg) produced reduction in pyrexia induced by turpentine. Significant (*p* < 0.05) effects observed with 200 mg/kg of extract were shown from 3 h Aspirin produced significant reduction of the pyrexia from 2 h (Figure 4).

#### d-Amphetamine-induced pyrexia

**Figure 5.**
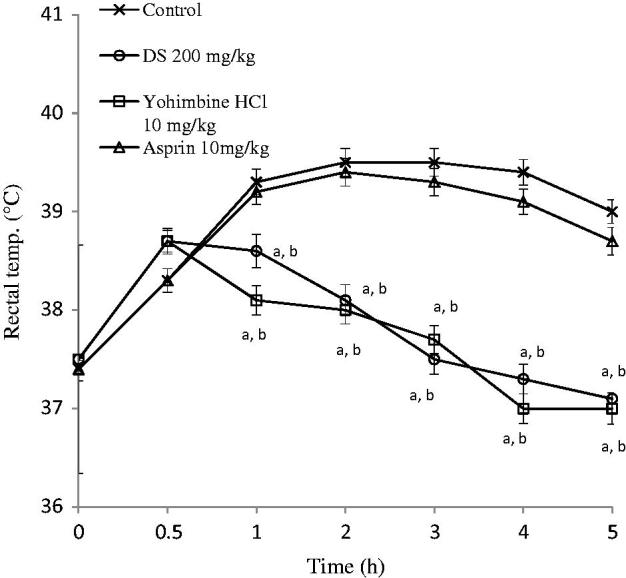
Graphs showing the effects of control (normal saline, 10 ml/kg), aqueous root extract of *Dalbergia saxatilis*, DS (200 mg/kg), yohimbine HCl (10 mg/kg) and aspirin (100 mg/kg) on pyrexia induced by D*-*amphetamine in rats. Significant (*p* < 0.05; ANOVA, Fisher’s *PLSD* test) reduction in rectal temperature compared with ^a^control given normal saline; ^b^10 mg/kg aspirin-treated animals at the same time; *n* = 5 per group.

Maximum pyrexia was observed 1 h after d*-*amphetamine sulphate was injected in control animals. The extract (100 and 200 mg/kg) produced significant (*p* < 0.05) reduction in pyrexia induced by d*-*amphetamine. These effects leading to hypothermia were observed from 0.5 h with 200 mg/kg of extract. Aspirin, however, did not significantly reduce the pyrexia (Figure 5).

## Discussion

In the analgesic model, DS, like aspirin, significantly inhibited the acetic acid-induced writhing in mice, a classical test useful for the evaluation of mild analgesic non-steroidal, anti-inflammatory drugs (NSAIDs) (Berkenkoff & Weichman 1988). This suggests that at least part of the analgesic action of the extract may be mediated peripherally like aspirin, a classical example of the NSAIDs. Aspirin is nonselective and irreversibly inhibits cyclooxygenase isozymes- COX-1 and COX-2 which produce prostaglandins, most of which are pro-inflammatory and cause pain (Sharma & Sharma 1997). However, the extract did not show effectiveness against the tail flick, tail immersion and hot plate as well as pressure (tail clip) pain tests. The tail flick, tail immersion and hot plate tests are acute thermic as well as phasic pain models, which have been considered to be selectively attenuated by centrally-acting opioid*-*like analgesic compounds (Chau 1989). It could therefore be suggested that DS possesses mild NSAID*-*like, but not opioid*-*like analgesic action. Furthermore, some analgesic effect was depicted in the first phase of the formalin test, but this efficacy was neither comparable to morphine nor aspirin. Subcutaneous injection of dilute formalin into mice hind-paw produces biphasic nociceptive response, namely: the first transient phase is caused by the direct effect of formalin on sensory C-fibers, and the second prolonged phase is associated with the development of the injury-induced spinal sensitization and ultimately central sensitization of the dorsal horn neuron occurring during inflammatory pain (Ashok et al. 2006). Drugs that act centrally, such as the narcotics, inhibit both phases of formalin-induced pain, while peripherally acting drugs such as aspirin only inhibit the late phases (da Ocha et al. 2011). Interestingly, the behaviour of DS did not elicit these biphasic postulations in the formalin test, thereby warranting further studies.

In the anti-inflammatory models, inhibition of carrageenan oedema was recorded only in the first hour of induction with the phlogistic agent. However, from the 2nd hour onwards the extract neither inhibited inflammation nor acted pro-inflammatory. Inflammatory action induced by carrageenan is known to be as a result of step-wise release of the inflammatory mediators such as histamine, serotonin and bradykinin which are released in the early phase of inflammatory reaction, and prostaglandins which are released late in the acute phase (Heller et al. 1998). It is probable that the active principles in DS act to inhibit histamine, serotonin and bradykinin in the first phase of inflammation, but might not act to either inhibit or cause the release of prostaglandins during tissue injury. Furthermore, the inhibitory effect of DS on dextran-induced inflammation, a model which is reported to be as a consequence of endogenous histamine and 5-HT release from mast cells, as well as kinins activation processes leading to osmotic oedema (Lo et al. 1982; Coura et al. 2015) confirmed probable anti-inflammatory activity. This action is a consequence of the ability of DS to inhibit endogenous histamine and serotonin release from the mast cells at the early phase of oedema. Also, recently, research findings have shown that during an inflammatory response, several other pro-inflammatory mediators are released, including interleukin-1β (IL-1β), tumour necrosis factor α (TNF-α) in addition to COX-2 (Coura et al. 2015). It is possible DS could also inhibit any of these. Nevertheless, the inhibitory effects by DS in these models were considerably lower than that of indomethacin, suggesting that DS possesses a minor anti-inflammatory action.

In the antipyretic test, DS produced significant and comparable effectiveness as aspirin in the *E. coli* lipopolysaccharide and turpentine-induced hyperthermia models. It has been reported that live and heat-killed Gram-negative (Toth & Krueger 1989) bacteria like *E. coli* elicit fever when administered to animals. An important pathway through which exogenous pyrogens such as lipopolysaccharides may induce fever is through direct induction of pro-inflammatory cytokine production by endothelial cells in the circumventricular organs (Netea et al. 2000). This suggests possible blocking of this cytokine production pathway by DS. The turpentine-induced hyperthermia is a rather neglected model, but with valuable pathophysiological mechanism of fever induction. This chemical had long been detected cause of pleural exudation (Bennett 1948), and that specifically, IL-1β seems to be the major mediator of fever after its injection (Horai et al. 1998). The significant effectiveness of DS in this model strongly indicates that its active principles could act by blocking interleukins, IL-1β, at least, for inflammation and fever. This is in addition to interference with the biosynthesis of cyclic prostanoids derived from arachidonic acid, such as thromboxane A_2_ and prostaglandins and some other steps involved in pyrexogenesis, like aspirin (Aronoff & Neilson 2001).

In the amphetamine model, DS and yohimbine, unlike aspirin, inhibited the hyperthermia induced by amphetamine, which suggests that this model might not involve the cyclooxygenase pathway. Yohimbine being an α_2_ adrenergic receptor blocker (Millan et al. 2000) therefore indicates that the adrenergic receptor mechanisms within the hypothalamus are involved in the fever induced by d-amphetamine. Interestingly, and comparably, yohimbine and DS significantly were effective in shortening the onset, lengthening the antipyretic duration, and being hypothermic below the basal body temperature at the end of 5 h in this model. d-Amphetamine pyrexia is reported to be due to increased metabolic heat production (Chi & Lin 1983). Since amphetamine is an indirectly-acting sympathomimetic, DS might have some antagonistic effect on endogenous catecholamines in the central adrenergic neurons, which supports the sedative actions previously reported for DS (Yemitan & Adeyemi 2003, 2005, 2013).

At this stage, the phytochemical(s) which is/are responsible for these pharmacological effects of DS may not be known, but the triterpenoid glycosides, one of which has been isolated as betulinic acid, might be involved. Further studies would ascertain this.

In conclusion, the aqueous root extract of *Dalbergia saxatilis* possesses mild non-steroidal analgesic and anti-inflammatory, but considerable antipyretic actions thought likely to be mediated through cyclo-oxygenase and IL-1β as well as central α-adrenoceptor blocking actions possibly due to the presence of any or combination of glycosides, saponins, and phenolic tannins as its active phytochemicals.
